# Gender differences in the association between anxiety symptoms and thyroid hormones in young patients with first-episode and drug naïve major depressive disorder

**DOI:** 10.3389/fpsyt.2023.1218551

**Published:** 2023-08-29

**Authors:** Ying Zhao, Jia Cheng Liu, Feng Yu, Li Ying Yang, Chuan Yi Kang, Li Juan Yan, Si Tong Liu, Na Zhao, Xiao Hong Wang, Xiang Yang Zhang

**Affiliations:** ^1^The Fourth Hospital of Harbin Medical University, Harbin, Heilongjiang Province, China; ^2^First Affiliated Hospital of Harbin Medical University, Harbin, Heilongjiang Province, China; ^3^Qingdao Mental Health Center, Qingdao, Shandong Province, China; ^4^Beijing Anding Hospital, Capital Medical University, Beijing, Beijing Municipality, China; ^5^Institute of Psychology, Chinese Academy of Sciences (CAS), Beijing, China; ^6^Department of Psychology, University of Chinese Academy of Sciences, Beijing, China

**Keywords:** major depressive disorder, anxiety, young, risk factor, gender differences

## Abstract

**Objective:**

Gender differences are prevalent in major depressive disorder (MDD), but the gender differences in the relationship between comorbid anxiety and thyroid hormones in young first-episode and drug-naive (FEND) MDD patients are unknown.

**Methods:**

A total of 1,289 young outpatients with FEDN MDD were recruited. Demographic and clinical data were collected for each patient. The patient’s blood glucose, blood pressure, thyroid hormone, and thyroid antibody levels were measured. The Hamilton depression scale (HAMD), Hamilton anxiety scale (HAMA), and Positive and Negative Syndrome Scale (PANSS) were used to assess patients’ depression, anxiety, and positive symptoms, respectively.

**Results:**

The prevalence of comorbid anxiety disorders was 80.4 and 79.4% in male and female MDD patients, respectively. Patients with anxiety had higher HAMD and PANSS scores, higher serum thyroid stimulating hormone (TSH), anti-thyroglobulin antibody (A-TG), and thyroid peroxidase antibody (A-TPO) levels, higher blood glucose and blood pressure levels, and more patients with psychotic symptoms and suicide attempts. Male patients were younger and had a younger age of onset. Logistic regression analysis showed that HAMD score and comorbid suicide attempts were significant predictors of anxiety symptoms in both males and females, whereas A-TG predicted anxiety symptoms in female patients only. Limitations: No causal relationship could be drawn due to the cross-sectional design.

**Conclusion:**

This study showed gender differences in factors associated with anxiety symptoms in patients with MDD. Some factors were associated with anxiety symptoms in both male and female patients, while A-TG was only associated with anxiety symptoms in female patients.

## 1. Introduction

Major depressive disorder (MDD) is one of the most common and debilitating psychiatric disorders, affecting more than 300 million people worldwide. Depression is the leading cause of suicide and is characterized by low mood, lack of motivation, diminished happiness, and sleep disturbances (1). Previous studies have shown that risk factors for MDD vary considerably from youth to adulthood to old age. MDD risk in young adults is more often expressed through psychological vulnerability and stress and genetic factors, whereas MDD risk in older adults is more often expressed through comorbid medical and neurological disorders (2, 3). In addition, anxiety disorders are a very common group of mental health conditions that can have a debilitating effect on daily functioning and well-being. People with anxiety disorders are also at higher risk for cardiovascular disease and premature death (4). They may co-occur with other mental health disorders, such as depression. It is known that 45–67% of people with MDD meet the criteria for at least one co-occurring anxiety disorder (5, 6). Similarly, 30–63% of patients with anxiety disorders meet criteria for co-occurring MDD (7–9). The prevalence of MDD with co-occurring anxiety disorders is approximately 11–80% (10–13). However, the incidence of anxiety in patients with MDD is highly heterogeneous across studies, which may be due to the different methods used to measure anxiety in these patients.

Anxious depression has a different neurobiological profile compared to non-anxious depression (5). Several studies have shown that anxious depression and non-anxious depression differ significantly in hypothalamic–pituitary-thyroid (HPT) axis function, structural and functional brain imaging findings, and inflammatory markers. Compared to non-anxious depression, patients with anxious depression have subtle changes in HPT parameters, i.e., alterations in thyroid antibodies and thyroid hormones (14). Furthermore, some symptoms of anxious depression (e.g., palpitations, increased sweating, fatigue, poor concentration, and sleep disturbances) overlap with the clinical manifestations of thyroid disorders (15). Previous studies have shown that the symptoms of patients with overt hypothyroidism overlap with those of MDD patients (16). However, controversy remains regarding thyroid changes in patients with anxiety disorders. One study found that hypothyroidism was more common in patients with anxiety disorders (17), while another study showed that hyperthyroidism was common in the same patients (18). Changes in thyroid hormone levels in patients with anxiety and depression remain to be further explored. Furthermore, patients with anxious depression are more likely to be present in primary care settings and are more likely to be associated with being female, non-single, unemployed, less educated, and more severely depressed (19, 20). Previous reports have also shown that patients with anxious depression have more frequent major depressive episodes and are at higher risk for suicidal ideation and previous suicide attempts compared to those with non-anxious depression (21, 22). Previous studies have also shown that patients with anxious depression is associated with variability in blood pressure, more likely to be associated with lower systolic blood pressure, with weaker or nonsignificant findings for diastolic blood pressure (23–25). The development of anxiety symptoms has also been associated with poor glycemic control (26). In addition, our previous study found that anxious patients with comorbid impaired fasting glucose were more likely to have problems with thyroid function, among other things, especially in female patients (27). The researchers found that patients with anxious depression had increased gray matter volume in the superior temporal gyrus and extended into the posterior middle temporal gyrus and inferior temporal gyrus of the right cerebral hemisphere compared to patients with non-anxious depression (28). These results imply that alterations in macrophage thickness in the overall pattern of brain sulci or gyrus structures may depend on the diagnosis (28). However, the mechanisms underlying MDD patients with anxiety are unclear and remain to be further investigated.

Women are known to be more likely than men to suffer from anxiety, dementia, panic disorder, post-traumatic stress disorder (PTSD), and MDD, although alcohol abuse is more common in men (29–31). Experimental data suggest that women are more susceptible to the deleterious effects of stress on mood and anxiety-related behaviors (32). Gender differences exist in the age of onset, progression, disease severity, underlying neuropathology, and response to treatment for neurological disorders, as well as significant gender differences, exist in brain physiology and behavior (33–35). Girls are more likely to suffer from depression than boys, and after adolescence, women’s susceptibility to anxiety increases to about twice that of men (36). This bias against females begins in adolescence and continues through middle age, roughly equivalent to the span of a woman’s childbearing years. Depressed women are more likely than men to experience anxiety, somatization, crying, anger, hostility, increased appetite, and weight gain (37). On the other hand, men exhibit lower self-esteem, more self-loathing, and mental depression than women. In addition, sex hormones affect depressive symptoms such as irritability, insomnia, appetite, and general physical health. In animal models, there are also strong indications that sexually differentiated behavior can be regulated by hormones (38). As with anxiety disorders, gender differences in symptom reporting and differential persistence may contribute to the high prevalence of depression in women (39). However, the increased prevalence of depression in women during perimenopause and menopause and in the postpartum period suggests that fluctuations in sex hormone levels play an important role in the susceptibility to depression and anxiety disorders in women (29).

Preclinical studies suggest that gender should be an important consideration when looking for potential biomarkers of depression (40). Women are more likely to suffer from depression and anxiety disorders than men, so they are treated with antidepressants to a greater extent (29). Careful consideration of gender differences in preclinical studies can facilitate and improve the design and quality of clinical studies of disease biomarkers and novel fast-acting antidepressants, which are critical for both men and women with depression (41, 42). Although it is well known that men and women have different susceptibilities to psychiatric disorders, gender is rarely considered when making diagnostic or treatment decisions. Therefore, a better understanding of the molecular basis behind these gender differences may help develop targeted therapies with higher success rates, especially in disorders where gender differences are most prominent. Previous studies have established that anxiety is a common co-occurring disorder in MDD and influences treatment outcomes in depression and that abnormal changes in thyroid hormones are present in both MDD and anxiety disorders (19, 43, 44). In addition, thyroid disease, although more common in older patients, is often masked by comorbidities (45). The increasing prevalence of thyroid disease and MDD in young adults necessitates research in this population (46, 47). However, few studies have explored gender differences in the relationship between comorbid anxiety and thyroid hormones in patients with first-episode and drug-naive (FEDN) MDD. To the best of our knowledge, our current study can be considered unique because it is the first study to examine gender differences in the relationship between anxiety and thyroid hormones in young FEDN MDD patients.

## 2. Methods

### 2.1. Participants and procedure

This was a cross-sectional study, and before the study started, the G-Power software calculated the minimum sample size of this study to be 512 individuals and considered a 20% miss visit rate. A total of 1,289 young FEDN MDD patients were recruited from the psychiatric outpatient clinic of the First Hospital of Shanxi Medical University in Shanxi Province, China, from 2015 to 2017. During the opening hours of the clinic (8:00 am to 6:00 pm), clinical psychiatrists were in charge of patient recruitment and scale assessment, and experienced nurses were in charge of blood collection. All participants were informed that it was their responsibility to decide or refuse to participate in this study and that they could withdraw at any time. Those patients who agreed to participate in our clinical study signed an informed consent form after a detailed explanation by the investigators. The Ethics Committee of the First Hospital of Shanxi Medical University approved the study, which was implemented in accordance with the Declaration of Helsinki.

All enrolled patients were required to meet the following criteria: (1) males and females between the ages of 18–45 years, Han Chinese; (2) meeting the diagnostic criteria for MDD in the Diagnostic and Statistical Manual of Mental Disorders, Fourth Edition (DSM-IV), and receiving follow-up within the following 3–6 months, with concordance between the two diagnoses; (3) first episode patients without prior drug treatment; and (4) scoring more than 24 on the 17-Hamilton Depression Rating Scale. Patients who met the following criteria were excluded: (1) severe physical illness; (2) history of substance or alcohol abuse, except for nicotine; (3) pregnancy or breastfeeding; (4) unable to understand or sign an informed consent form; and (5) self-identified as non-binary or transgender.

### 2.2. Assessment and measurement process

A self-administered questionnaire was used to collect sociodemographic information, including age, duration of illness, age of onset, and marital status. In our study, the 17-item Hamilton Assessment Scale for Depression (HAMD) was used to assess levels of depression (48). The 17-item HAMD scale contains eight items on a 5-point scale (0: not present, 4: severe) and nine items on a 3-point scale (0: not present, 2: severe). The presence and the severity of depression are determined based on the HAMD total score.

In our study, the 14-item Hamilton Anxiety Rating Scale (HAMA) was used to assess anxiety levels (49). The HAMA scale consists of 14 items, all of which are scored on a 5-point scale (0: not present, 4: severe). The presence and degree of anxiety are determined based on the HAMA total score. Patients were considered to have severe anxiety symptoms if their total score reached or exceeded 18 (50).

The positive subscale of the Positive and Negative Syndrome Scale (PANSS) was used to assess the severity of patients’ psychotic symptoms (51). The positive subscale of the PANSS consists of seven items, each of which is scored on a 7-point scale (1: not present, 7: extremely serious). In our study, a score of 15 was used as a cut-off value to determine whether the patient had psychotic symptoms (51). Thus, when the score was greater than or equal to 15, the patient was determined to have psychotic symptoms.

Definition of suicide attempt: in this study, suicide attempt was defined as any act of self-harm by the participant to end his/her life (52, 53). Suicide attempts were conducted through interviews. The participant was asked, “Have you ever attempted suicide in your life?” This question was taken from the WHO/EURO multicenter study (54). If the answer was yes, he/she was categorized as a suicide attempter. Otherwise, he/she was a non-suicide attempter. In addition, we investigated information about the frequency, method, and exact date of suicide attempts. If their answers were vague, the researchers clarified this information by interviewing their family members, relatives, or friends. Further intervention is necessary for patients who have made suicide attempts. We have advised their guardians to provide intensive 24-h care and to stay away from suicide tools and places.

In the present study, the two psychiatrists who collected the above information attended training using HAMD, HAMA, and PANSS. They were blinded to the subject’s clinical data beforehand. Repeated assessments showed that the correlation coefficient between raters of HAMD, HAMA, and PANSS total scores remained above 0.8.

### 2.3. Physical and biochemical parameters measurements

The researchers collected fasting venous blood from the participants between 6 and 8 am. All blood samples were immediately sent to the testing center of the First Clinical Medical College of Shanxi Medical University and were tested by 11:00 am on the same day. The biochemical indicators collected in this study included thyroid stimulating hormone (TSH), anti-thyroglobulin antibody (A-TG), thyroid peroxidase antibody (A-TPO), free triiodothyronine (FT3), and free thyroxine (FT4). The normal range of TSH is 0.27–4.20 mIU/L, A-TG is 0–115 IU/L, A-TPO is 0–34 IU/L, FT3 is 3.10–6.8 pmol/L, and FT4 is 10–23 pmol/L. Above or below normal levels of the above indicators were considered abnormal changes and were judged normal if they were within the normal range. In addition, the researchers monitored the participants’ systolic blood pressure (SBP), diastolic blood pressure (DBP), body mass index (BMI), and fasting blood glucose (FBG).

### 2.4. Statistical analysis

Our study used SPSS 23.0 statistical software for data analysis. Data were described as mean ± standard deviation (SD) or percentages (%). The Kolmogorov–Smirnov (K-S) test was used to test whether the data conformed to a normal distribution (*p* > 0.05). Analysis of variance (ANOVA) was performed for variables that conformed to a normal distribution, and nonparametric tests were performed for variables that did not conform to a normal distribution. ANOVA or nonparametric tests were performed for continuous variables, and chi-square tests were performed for categorical variables to compare demographic and clinically relevant variables between male and female patients. To compare gender differences between patients’ comorbid anxiety symptoms and clinical parameters, this study used a 2 × 2 ANOVA, considering both diagnosis (2 levels: with severe anxiety and without severe anxiety) and gender (2 levels: male and female). The main effects of gender and with or without anxiety subgroups, as well as the interaction between gender × with or without anxiety subgroups, were tested. Correlations between HAMA score and demographic and clinical variables were assessed by Pearson or Spearman correlation coefficients for male and female patients as appropriate, respectively.

To analyze the presence of independent risk factors for anxiety in men and women with MDD. Univariate analyses were performed for male and female MDD patients separately, using anxiety as the dependent variable and sociodemographic and clinical variables as covariates. Finally, indicators that changed significantly in the univariate analysis were included in a binary logistic regression model. Bonferroni correction was performed for multiple tests. In our study, the significant value of p was set at 0.05 (two-tailed).

## 3. Results

### 3.1. Gender differences in demographic and clinical characteristics of young FEDN MDD patients

This study enrolled 1,289 patients according to the inclusion criteria. Of these, 470 were male and 819 were female. As shown in Table 1, we found that age (*F* = 6.261, *p* = 0.012) and age of onset (*F* = 6.422, *p* = 0.011) were greater in female patients than in male patients. In the present study, the mean age of female patients was 29.74 years and that of male patients was 28.49 years. The mean age at onset was 29.59 for female patients and 28.33 years for male patients. This shows that the age and age of onset were below 30 years for both sexes. In addition, we did not find gender differences in variables such as HAMD score, positive symptom score, and thyroid hormone levels (all *p*>0.05).

**Table 1:** Demographic and clinical characteristics between young FEDN MDD patients with and without anxiety, grouped by gender (*n* = 1,289).

	MDD without Anxiety (*n* = 261)		MDD with Anxiety (*n* = 1,028)		Anxiety *F*/ *χ*^2^ (*p*)	Gender F/ χ^2^ (*p*)	Anxiety X Gender *F*/ *χ*^2^ (*p*)
	Male	Female	Male	Female			
Sample size	92	169	378	650			
Age (years)	27.16 (7.99)	30.78 (9.12)	28.81 (8.78)	29.48 (8.54)	0.202 (0.653)	6.261 (0.012)	5.543 (0.019)
Duration of illness (month)	5.57 (4.55)	5.83 (4.39)	5.67 (4.15)	5.61 (3.93)	0.141 (0.707)	0.000 (0.986)	0.299 (0.584)
Age of onset (years)	27.00(7.86)	30.58(9.05)	28.66(8.69)	29.33(8.44)	0.152(0.696)	6.422(0.011)	5,429(0.019)
HAMD	28.01 (2.23)	28.16 (2.43)	30.70 (2.86)	30.71 (2.78)	189.690 (<0.001)	0.007 (0.932)	0.114 (0.735)
Positive symptoms	7.04 (0.417)	7.12 (1.15)	8.75 (4.05)	9.13 (4.57)	47.991 (<0.001)	1.725 (0.189)	0.286 (0.593)
TSH	4.18 (1.72)	4.10 (1.69)	5.09 (2.59)	5.18 (2.70)	34.755 (<0.001)	0.125 (0.724)	0.239 (0.625)
FT3	4.99 (0.72)	4.83 (0.79)	4.96 (0.74)	4.91 (0.71)	0.524 (0.496)	2.768 (0.096)	0.854 (0.355)
FT4	16.89 (3.19)	16.68 (2.91)	16.74 (3.18)	16.75 (3.05)	0.001 (0.971)	0.024 (0.877)	0.263 (0.608)
A-TG	54.46 (111.75)	33.90 (55.11)	84.38 (185.86)	101.78 (258.57)	13.548 (<0.001)	0.560 (0.454)	1.521 (0.218)
A-TPO	35.24 (84.81)	36.76 (95.55)	78.66 (167.76)	77.05 (172.31)	14.331 (<0.001)	0.024 (0.877)	0.019 (0.891)
BMI (kg/m^2^)	24.22 (2.04)	24.49 (1.69)	24.43 (2.12)	24.28 (1.90)	0.187 (0.666)	0.308 (0.597)	2.245 (0.134)
FBG	5.24 (0.55)	5.28 (0.58)	5.38 (0.65)	5.41 (0.65)	9.887 (0.002)	1.145 (0.285)	0.006 (0.940)
Systolic pressure	113.75 (9.12)	114.98 (9.96)	117.34 (10.152)	117.25 (10.75)	14.539 (<0.001)	0.057 (0.812)	0.778 (0.378)
Diastolic pressure	73.40 (5.906)	74.03 (5.92)	75.90 (6.62)	75.64 (6.04)	20.193 (<0.001)	0.085 (0.771)	0.984 (0.321)
Unmarried, N(%)	41 (44.6%)	60 (35.5%)	151 (39.9%)	235 (36.2%)	0.117 (0.732)	2.966 (0.085)	N
With psychotic symptoms	0 (0%)	1 (0.6%)	33 (8.7%)	73 (11.2%)	26.954 (<0.001)	1.592 (0.207)	N
With Suicidal behavior	3 (3.3%)	4 (2.4%)	79 (20.9%)	158 (24.3%)	56.295 (<0.001)	1.060 (0.303)	N

HAMD, hamilton depression rating scale; TSH, thyroid stimulating Hormone; FT3, free triiodothyronine; FT4, free thyroxine; A-TG, anti-thyroglobulin antibodies; A-TPO, thyroid peroxidases antibody; BMI, body mass index; FBG, fasting blood glucose. N: no variable was not included in the analysis becasuse they were not eligible.

### 3.2. Gender differences in demographic and clinical characteristics of young FEDN MDD patients with and without anxiety

Of the 1,289 patients in this study, 1,028 were in the comorbid anxiety group and 261 were in the non-comorbid anxiety group. As shown in Table 1, the prevalence of comorbid anxiety among patients with FEDN MDD was 79.75% (1028/1289). There was no significant sex difference in the prevalence of comorbid anxiety in MDD patients (80.4% for males and 79.4% for females).

In this study, the interaction between anxiety and gender was calculated using ANOVA. A two-way ANOVA combining the between-group factors of anxiety and gender showed that anxiety had a significant effect on HAMD total score (*F* = 189.690, *p* < 0.001), positive symptom score (*F* = 47.991, *p* < 0.001), TSH (*F* = 34.755, *p* < 0.001), A-TG (*F* = 13.548, *p* < 0.001), A-TPO (*F* = 14. 331, *p* < 0.001), FBG (*F* = 9.887, *p* = 0.002), systolic blood pressure (*F* = 14.539, *p* < 0.001), diastolic blood pressure (*F* = 20.193, *p* < 0.001), psychotic symptoms (2 = 26. 954, *p* < 0.001), and suicide attempts (2 = 56.295, *p* < 0.001). Gender had a significant effect on age (*F* = 6.261, *p* = 0.012) and age of onset (*F* = 6.422, *p* = 0.011). In addition, we found an interaction between anxiety and gender on age (*F* = 5.543, *p* = 0.019) and age of onset (*F* = 5.429, *p* = 0.019). Taken together, this study found gender differences in certain clinical characteristics between MDD patients with and without anxiety.

### 3.3. Correlation of HAMA score with demographic and clinical variables in male and female patients with FEND MDD

The results of the correlates of comorbid anxiety in patients with FEDN MDD are shown in Table 2. In the male group, anxiety was associated with HAMD total score (*r* = 0.618, *p* < 0.001), positive symptom score (*r* = 0.592, *p* < 0.001), TSH (*r* = 0.290, *p* < 0. 001), A-TG (*r* = 0.164, *p* < 0.001), A-TPO (*r* = 0.213, *p* < 0.001), FBG (*r* = 0.095, *p* = 0.041), systolic blood pressure (*r* = 0.177, *p* < 0.001), and diastolic blood pressure (*r* = 0.170, *p* < 0.001). However, the significance in FBG did not pass the Bonferroni correction (*p* > 0.05/14 = 0.004).

**Table 2:** Correlation of HAMA scores with demographic and clinical variables in male and female FEDN MDD patients.

	Male		Female	
	*r*	*p*	*r*	*p*
Age (years)	0.073	0.112	−0.035	0.320
Duration of illness (month)	0.025	0.584	0.017	0.619
Age of onset (years)	0.074	0.107	−0.034	0.327
HAMD	0.618	<0.001	0.585	<0.001
Positive symptoms	0.592	<0.001	0.572	<0.001
TSH	0.290	<0.001	0.333	<0.001
FT3	−0.073	0.112	0.037	0.294
FT4	0.034	0.468	0.018	0.599
A-TG	0.164	<0.001	0.105	0.003
A-TPO	0.213	<0.001	0.094	0.007
BMI (kg/m^2^)	0.027	0.562	0.016	0.639
FBG	0.095	0.041	0.125	<0.001
Systolic pressure	0.177	<0.001	0.187	<0.001
Diastolic pressure	0.170	<0.001	0.190	<0.001

HAMD, hamilton depression rating scale; TSH, thyroid stimulating hormone; FT3, free triiodothyronine; FT4, free thyroxine; A-TG, anti-thyroglobulin antibodies; A-TPO, thyroid peroxidases antibody; BMI, body mass index; FBG, fasting blood glucose.

In the female group, anxiety was associated with HAMD total score (*r* = 0.585, *p* < 0.001), positive symptom score (*r* = 0.572, *p* < 0.001), TSH (*r* = 0.333, *p* < 0.001), A-TG (*r* = 0.105, *p* = 0.003), A-TPO (*r* = 0.094, *p* = 0.007), FBG (*r* = 0.125, *p* < 0.001), systolic blood pressure (*r* = 0.187, *p* < 0.001), and diastolic blood pressure (*r* = 0.190, *p* < 0.001). After Bonferroni correction, these differences remained significant (all *p* < 0.05/14 = 0.004). These results suggest that the statistically significant variables were positively correlated with the HAMA scores for both male and female patients, i.e., the higher the levels of these variables, the more severe the anxiety.

### 3.4. Binary logistic regression analysis of factors influencing anxiety in male and female patients with FEND MDD

The results of independent risk factors for anxiety in the male and female groups are shown in Table 3. In the male group, both HAMD total score (OR = 1.482, 95% CI = 1.295–1.696, *p* < 0.001) and suicide attempts (OR = 4.280, 95% CI = 1.234–14.843, *p* = 0.022) were independent risk factors for anxiety. In the female group, HAMD total score (OR = 1.327, 95% CI = 1.206–1.460, *p* < 0.001), suicide attempts (OR = 6.619, 95% CI = 2.235–18.846, *p* < 0.001), and A-TG levels (OR = 1.004, 95% CI = 1.001–1.008, *p* = 0.008) were all independent risk factors for anxiety.

**Table 3:** Demographic and clinical variables independently associated with anxiety in each gender.

Variables	Male					Female				
	B	Wald statistic	*p*	OR	95% CI	B	Wald statistic	*p*	OR	95% CI
HAMD	0.394	32.681	<0.001	1.482	1.295–1.696	0.283	33.666	<0.001	1.327	1.206–1.460
With suicidal behavior	1.454	5.252	0.022	4.280	1.234–14.843	1.890	12.534	<0.001	6.619	2.325–18.846
A-TG	N	N	N	N	N	0.004	7.100	0.008	1.004	1.001–1.008

HAMD, hamilton depression rating scale; TSH, thyroid stimulating hormone; A-TG, anti-thyroglobulin antibodies. N: no variable was not included in the analysis becasuse they were not eligible.

## 4. Discussion

To our knowledge, the present study is the first to examine gender differences in the relationship between comorbid anxiety and thyroid hormones in young patients with FEDN MDD. Our findings showed that (1) there were no gender differences in the prevalence of comorbid anxiety in patients with FEDN MDD, with high prevalence in both men and women (80.4% vs. 79. 4%); (2) compared with patients without anxiety, patients with anxiety had more severe depression, higher positive symptom score, higher TSH, A-TG and A-TPO levels, higher blood pressure, higher blood glucose levels, and were more likely to have psychotic symptoms and suicide attempts; and (3) there were gender differences in the correlation between anxiety symptoms and demographic and clinical variables in patients with FEDN MDD.

Our study found that anxiety was 80.4 and 79.4% in men and women, respectively. Previous studies have found high rates of comorbidity between anxiety and depression (50–80%) and that anxiety is often a precursor to depression (55). Most depressed adolescents appear to suffer from psychiatric disorders throughout their lives, with the highest rates of comorbid anxiety at 30–80% (56). These studies are generally consistent with our findings. Several studies have investigated differences in the prevalence of comorbid anxiety disorders between males and females, with inconsistent results. For example, one study found that the prevalence of comorbid anxiety was higher in women than in men with depression (57). Another survey of older Asian patients with depression showed that the prevalence of comorbid anxiety disorders was 16.84%, and the comorbidity rate of anxiety disorders was associated with women (58). A recent study found that 18.3% of college students had comorbid anxiety and depressive symptoms. Meanwhile, there was no statistically significant difference between males and females in terms of comorbid anxiety and depressive symptoms (59). Our study found no difference in the prevalence of anxiety among MDD patients between males and females. The inconsistency with the results of the above studies may be the different ages of the selected patients. Some studies focused on older adults or adolescents. In addition, the study samples differed. We targeted younger patients with FEND MDD, which may explain the higher prevalence of anxiety and the lack of gender differences.

The results of our study showed that the age and age of onset of MDD were higher in women than in men (Table 1). In contrast to our results, data from Western countries suggest that the age of onset of MDD in women is younger (60, 61) or not different from men (37, 62). Regional and economic differences may have contributed to the different results of the study. Furthermore, it is known that anxiety is an adaptive human experience that can occur at all ages (63). The present study found an interaction between anxiety and gender in terms of age and age of onset (Table 1). Thus, crosstalk between anxiety and age may also have contributed to the different results of the study. Indeed, data from middle or low-income countries show that the prevalence of depression and anxiety increases with age (64, 65). In any case, timely screening of young people for depression and anxiety remains critical. In addition, this study found that blood pressure and blood glucose levels were higher in patients with anxiety compared to those without anxiety (Table 1). This is in line with previous findings on the effect of blood glucose (66) or blood pressure (67) on anxiety. Measuring these convenient biological indicators would certainly be helpful for the timely detection of anxiety symptoms in patients with MDD in our clinical, community, or home settings. In addition, this study found that patients with anxiety had more psychotic symptoms compared to those without anxiety (Table 1). Both anxiety and depression have been reported to be associated with the severity of psychotic symptoms (68). A study of 1718 patients with MDD found that patients with anxiety had higher psychotic symptoms than those without severe anxiety symptoms, supporting our results (69).

This study found that patients with anxiety had more suicide attempts and higher HAMD scores compared to those without anxiety (Table 1). Further logistic regression confirmed that suicide attempts and HAMD scores were independent risk factors for anxiety symptoms in young FEDN MDD patients (Table 3). It is well known that patients with MDD are at high risk of suicide (70). Anxiety disorders often coexist with MDD and may increase the risk of suicide (71, 72). A UK study of older people with MDD showed that one in six depressed people who committed suicide were identified as having anxiety disorders and were associated with multiple suicide risk factors (73). A systematic review showed that comorbid anxiety and higher levels of depression were significant correlates of suicide in people with MDD (74). In addition, there is considerable evidence supporting the association of depression severity with the development of anxiety disorders, supporting our results (75–78).

Thyroglobulin autoantibodies (A-TG) and thyroid peroxidase autoantibodies (A-TPO) are markers of thyroid autoimmunity (79). Previous studies have proposed that high levels of thyroid autoantibodies are associated with an increased frequency of mood disorders observed in thyroid disease (80, 81). Currently, there are more studies on A-TPO and few studies focusing on A-TG. An important finding of this study was that anxiety symptoms and thyroid hormone and antibody levels were correlated in younger patients with FEDN MDD, and there were gender differences. The results of this study showed that serum TSH, A-TG, and A-TPO levels were higher in anxious patients compared to those without anxiety (Table 1). Further logistic regression confirmed that serum A-TG was an independent risk factor for comorbid anxiety in female patients (Table 3). Indeed, thyroid-related disorders (e.g., hypo- and hyperthyroidism) are more common in women than men (82). Previous studies have shown that depressed patients frequently present with thyroid dysfunction, manifested as an increase or decrease in thyroid hormones (83). In addition, thyroid dysfunction has been associated with the onset of anxiety. However, this is an inconsistent finding. In contrast to the results of the present study, a survey of 2,142 patients found that hypothyroidism was positively associated with depression and anxiety, hyperthyroidism was significantly associated with MDD, while serum TSH levels and A-TPO were not significantly associated with depression and anxiety (43). Similar to the results of the present study, several studies have noted that higher serum A-TPO levels in patients with anxiety disorders are positively associated with anxiety symptoms (84, 85). In addition, our recent study found that patients with psychotic MDD with severe anxiety had higher serum TSH, A-TG, and A-TPO levels compared to patients with psychotic MDD without severe anxiety (86). Differences in the target population and the number of comorbidities may account for these disparities. Regarding the gender differences in serum A-TG in patients with comorbid anxiety disorders found in this study, this may be related to the gender dimorphism present in the immune response of men and women and the involvement of sex hormones, which have been shown in previous studies to be responsible for the higher thyroid autoimmunity in women (87, 88). In addition, the role of thyroid hormones in the male and female brain, as well as different key genes and signaling networks, may also be responsible for this difference (89). Therefore, the mechanism of the effect of A-TG on anxiety in female patients should still be further investigated.

The limitations of the present study are as follows. First, because of the cross-sectional design, the present study could not draw a causal relationship between anxiety and related factors. Our results need to be confirmed by a larger prospective cohort study. Second, the patients recruited for this study were from outpatient clinics and may not be generalizable to inpatients and community patients. Our results need to be tested in a different clinical setting. Third, relatively few variables were collected in this study; for example, data on personality traits, comorbidities, and family history related to anxiety were not collected. Fourth, we used a clinical interview to assess suicide attempts without using a structured instrument, which may have led to the omission of some patients’ existing suicidal ideation. Fifth, this study only investigated thyroid hormone levels, and follow-up research should continue to explore the relationship between thyroid-related disorders and anxiety symptoms in young MDD patients.

Overall, this study showed a very high prevalence of anxiety in both male and female young FEDN MDD patients. The severity of depression and concomitant suicide attempts were risk factors for the development of anxiety in both males and females. In addition, gender differences related to the occurrence of anxiety were observed in this study. This was mainly reflected in the fact that A-TG was an independent risk factor for the development of anxiety in female patients. Although these results require further study, this study remains critical for clinicians to target the identification of high-risk groups and to regularly screen for relevant clinical indicators to reduce the occurrence of anxiety.

## Data availability statement

The original contributions presented in the study are included in the article/supplementary materials, further inquiries can be directed to the corresponding author.

## Ethics statement

The studies involving humans were approved by The Ethics Committee of the First Hospital of Shanxi Medical University. The studies were conducted in accordance with the local legislation and institutional requirements. The participants provided their written informed consent to participate in this study.

## Author contributions

YZ, JL, FY, LY, NZ, XW, and XZ were responsible for study design, statistical analysis, and manuscript preparation. CK, LY, and SL were accountable for the data curation. XZ, NZ, and XW were involved in evolving the ideas and editing the manuscript. All authors contributed to the article and approved the submitted version.

## Funding

This work was supported by the Natural Science Foundation of Heilongjiang Province (LH2022H040) and the First Affiliated Hospital of Harbin Medical University (2021B22). These funders had no role in the design and conduct of the study, collection, analysis, interpretation of the data, and preparation, review, or approval of the manuscript.

## Conflict of interest

The authors declare that the research was conducted in the absence of any commercial or financial relationships that could be construed as a potential conflict of interest.

## Publisher’s note

All claims expressed in this article are solely those of the authors and do not necessarily represent those of their affiliated organizations, or those of the publisher, the editors and the reviewers. Any product that may be evaluated in this article, or claim that may be made by its manufacturer, is not guaranteed or endorsed by the publisher.
